# Is partisan conflict over COVID-19 vaccination eroding support for childhood vaccine mandates?

**DOI:** 10.1038/s41541-023-00611-3

**Published:** 2023-02-02

**Authors:** Matt Motta

**Affiliations:** Department of Health Law, Policy, & Management, Boston University School of Public Health, Boston, MA USA

**Keywords:** Public health, Infectious diseases

Americans’ partisan identity (i.e., the political party with which they identify) has become one of the primary determinants of whether or not individuals opt to vaccinate against COVID-19^1^. Partisan differences in vaccine uptake are likely driven by disagreement about vaccine safety and efficacy^2,3^, which may result from asymmetric exposure to vaccine misinformation and efforts to downplay pandemic severity among Republican politicians and partisan news outlets^1,4–7^.

Correspondingly, researchers^8^, and journalists^9,10^ alike have expressed concern that negative attitudes toward the COVID vaccine one the ideological right could “spill over” to shape Americans’ views toward other vaccines. A collection of recent polls found, for example, that whereas Democrats and Republicans tended to vaccinate at roughly equal rates prior to the onset of the pandemic, Republicans have become less likely to do so following COVID-19 vaccine rollout^9^.

## Politicized COVID-19 vaccine spillover could challenge evidence-based vaccine policy

Beyond politicizing Americans’ vaccine attitudes and behaviors, COVID-19 vaccine spillover could also have *problematic health policy consequences*. Republicans who have come to hold more negative views toward vaccines may express support for policies that loosen or remove altogether policies that encourage widespread vaccination. In theory, this creates an electoral incentive for policymakers to respond to public demands for weakened vaccine policies^11^.

State lawmakers already appear to be responding to politicized COVID-19 vaccine spillover, in the general public^12,13^. For example, Ron Nate, a Republican lawmaker from Idaho, introduced legislation in Fall 2021 that would redefine *all vaccine mandates* – not just COVID-19 vaccine mandates subject to legal challenges by dozens of Republican attorneys general across the country – as a form of “assault.” Nate was joined by several other state politicians who introduced similar legislation in direct response to fears of government overreach resulting from the Biden administration’s efforts to require COVID-19 vaccination in certain employment settings^14^.

While Nate’s legislation was not ultimately passed into law, it may only be a matter of time before vaccine mandates – including requirements that children are vaccinated against illnesses like Measles, Mumps, and Rubella (i.e., via the MMR vaccine) – are passed into law. Critical in determining whether or not this is the case is the degree to which experiences with the COVID-19 pandemic and roll out of a vaccine have led the public to express a *demand* for relaxed vaccine policies.

## Evidence of Politicized COVID-19 Vaccine Spillover

Evidence of COVID-19 vaccine spillover to childhood vaccine mandates is scarce. My goal in this commentary, then, is two-fold.

First, I want to offer new (albeit incomplete) evidence suggesting that negative views toward COVID-19 vaccines may be shaping public opinion toward MMR vaccination. Second, I hope to outline a path by which scholars can better understand whether or not politicized vaccine spillover is occurring, and discuss how this research can inform efforts to preserve policies that foster vaccination.

Regarding the former, scholars are yet to explore how politicized opposition to COVID-19 vaccination may be encouraging people to hold more negative views toward childhood vaccine mandates. I offer some preliminary evidence that this might be occurring by turning to data from the Vaccine Adverse Event Reporting System (VAERS).

VAERS is a federally run database that registers reports of negative side effects resulting from vaccination. While VAERS data are useful in helping public health experts anticipate potential vaccine-related complications, these reports are not validated upon their initial submission. We do not necessarily know that individuals reporting side effects *actually* experienced those harms.

Correspondingly, recent research^15,16^ suggests that VAERS data may function as an indicator of *public vaccine sentiment*. While VAERS data cannot tell us precisely how many Americans hold positive or negative views toward vaccination, increased side effect reporting may be indicative of increased public fears about vaccine safety. Our past research^15^ validates the use of VAERS data to this end by showing that, whereas MMR side effect reports were relatively rare prior to the publication of a controversial study alleging a link between childhood vaccination and autism, adverse event reports spiked dramatically following the publication of this report and media attention to its fraudulent claims.

**Fig. 1:**
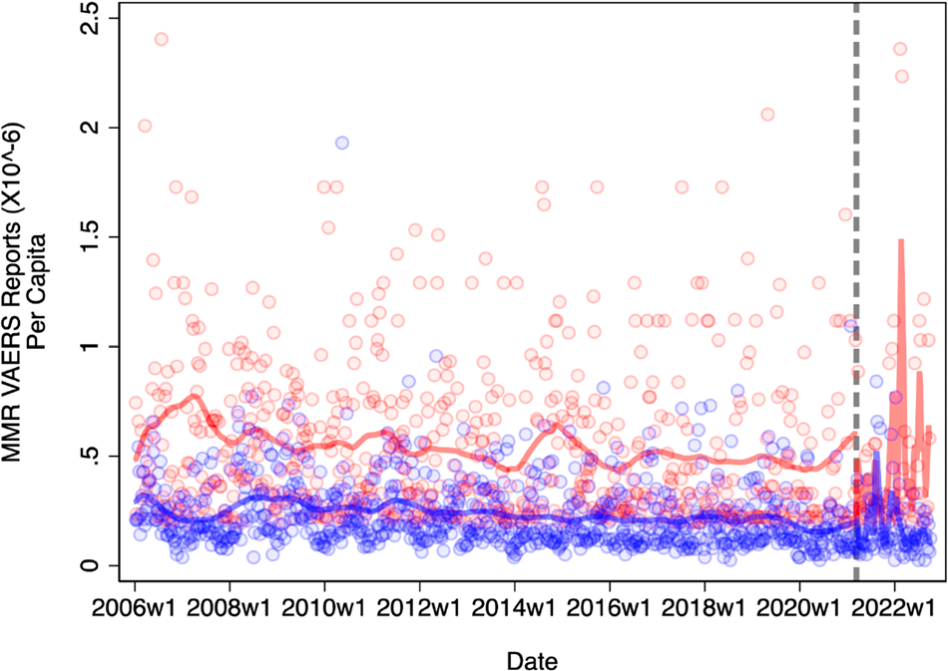
Weekly Per Capita MMR and MMRV Adverse Event Reports Averaged Across Democratic-Leaning and Republican-Leaning States (1/06 - 10/22). Please see the Online Supplement for detailed information about the measures and analytical procedures used to construct this figure, and its corresponding significance tests. Blue and Red dots correspond to weekly adverse event reports logged across Democratic-leaning and Republican-leaning states (respectively); operationalized as the eight states with the strongest records of voting for Republican and Democratic candidates in 2022 (NB: sixteen total states are included in this analysis). Blue and red lines are locally weighted regression lines (bandwidth = 0.10) calculated between each of these points. Weekly VAERS reports are averaged across states, and scaled to account for differences in state-level population. For ease of visual interpretation, those quantities are multiplied by a factor of 10^6^. The dashed gray line indicates the start of March 2021; i.e., an estimate of the general timeframe in which COVID-19 vaccines started to become widely available for American adults beyond those belonging to high-risk populations. Four formal regression discontinuity analyses are available in the online supplement, and suggest that reporting differences between blue and red states were significantly larger in the post-vaccine availability period (*p* > 0.10 two-tailed in just one out of four tests). Outlying observations are trimmed from visual display at 2.5, but are included in the calculation of locally weighted regression lines and regression discontinuity results.

Figure 1 adopts this approach to present preliminary evidence of politicized COVID-19 vaccine spillover to MMR vaccine sentiment. The figure’s y axis denotes the *per capita* adverse event reporting rate in the eight most Democratic-leaning (blue line) versus eight most Republican-leaning (red line) states. I defined states’ partisan lean via a widely-used measure of state-level partisanship known as the Partisan Voting Index^17^. Additional information about this measure and procedure can be found in the online supplement.

The x axis corresponds to average weekly adverse event reports for two commonly-administered childhood vaccines (MMR and MRRV; pooled) from 2006 (the first year in which both MMR and MMRV were approved for public use) through Fall 2022, across blue and red states. The dashed gray line denotes March 2021, i.e., the first month in which states began to open COVID-19 vaccination to the general public, beyond smaller at-risk groups.

If negative views toward the COVID-19 vaccine in red states are depressing public confidence in MMR vaccination, we should expect to see a spike in adverse event reports in red states, but *not* blue states, following vaccine roll-out. Indeed, this is precisely what I find. While red states were more likely to register adverse event reports than blue states prior to the availability of a COVID-19 vaccine, these differences spiked dramatically following vaccine rollout. Supplementary regression discontinuity analyses suggest that the difference in reports logged between blue and red states was significantly larger (at the *p* < 0.05 in one of four tests and at the *p* < 0.10 level in two out of four tests) following the availability of a COVID-19 vaccine than it was previously. Additional details about these analyses can be found in the online supplement.

Of course, these data do not permit me to investigate how many Americans hold negative views toward MMR vaccines, or vaccine mandates. They also don’t allow me to assess how individuals may have come to update their views over time, and the degree to which concerns about the COVID-19 vaccine (specifically) are responsible for these effects.

## Going forward: a research strategy for assessing politicized COVID-19 vaccine spillover

Correspondingly, the public health community ought to engage in research practices aimed at identifying whether or not politicized COVID-19 vaccine attitudes are shaping public opposition to childhood vaccine mandates.

To do this, I argue that scholars ought to pursue some combination of the following three research designs. While these strategies are likely best employed *together* (i.e., in service of providing methodologically pluralistic assessments of spillover potential), I order my discussion of each one with respect to how well-equipped they are to draw valid inferences about potential spillover effects.

### Longitudinal assessments of politicized vaccine spillover

Perhaps the most powerful way that scholars can determine whether or not COVID-19 vaccine attitudes are shaping how Americans view other vaccines is to conduct longitudinal survey research that asks *the same group of people, over time*, to report their attitudes toward both COVID-19 vaccination and vaccine mandates more generally. If people who come to hold more negative attitudes toward the COVID vaccine over time are also more likely to revise previously-held support or indifference about the acceptability of vaccine mandates, we will have documented politicized spillover effects. One major benefit of longitudinal designs is that, because they are concerned with documenting within-person change, they lend themselves well to statistical modeling strategies that estimate opinion change that is independent of between-person differences; thereby implying that potential between-person confounds (such as survey respondents’ age, race, and/or educational attainment) cannot possibly explain why we might observe evidence of spillover. One challenge, however, is that efforts to survey Americans’ vaccine-related attitudes toward vaccination *before* the availability of COVID-19 vaccines may not exist. Making an effort to incentivize recontact those respondents included in surveys that ask these questions pre-COVID is therefore of paramount importance for the viability of this method.

### Experimental assessments of politicized vaccine spillover

Scholars can also assess spillover by conducting randomized controlled trials (RCTs) that randomly encourage some people (but not others) to think about COVID-19 vaccination prior to answering questions about childhood vaccine mandates. This might take the form, for example, of a “thought listing task”^18–21^ that encourages people to call to mind positive and/or negative views toward the COVID-19 vaccine prior to rendering judgments about the suitability of vaccine mandates more generally. If individuals who express negative views (who may be disproportionately likely to self-identify as Republicans) are then more likely to oppose vaccine mandates more generally, we will again have evidence of spillover. As noted above, a key benefit of RCT efforts to detect spillover is that – because subjects are randomly exposed to primes that encourage them to think about the COVID-19 vaccine – any spillover effects we might document are not likely to be subject to potential between-person confounds. A potential drawback, however, is that while experimental approaches can convincingly tell us whether or not spillover occurs, external validity constraints may make RCTs less well-suited to tell us *how much* spillover occurs. For example, because most people do not generate lists of arguments for/against vaccination on a regular basis in their daily lives, thought-listing tasks in RCTs could over- or underestimate spillover effect prevalence.

### Observational assessments of politicized vaccine spillover

Finally, scholars might consider studying observationally whether or not attitudes toward COVID-19 vaccination are correlated with views toward childhood vaccine mandates. Correlational analyses are, necessarily, potentially confounded by between-person factors that might influence views toward vaccination, and cannot disentangle whether or not attitudes toward COVID-19 vaccination *antecede* those of vaccine mandates more generally. It is therefore critically important that scholars both theoretically identify and empirically measure as many potential attitudinal (e.g., political and religious views), psychological (e.g., aversions to needles, preferences for bodily purity), health-economic (e.g., health insurance coverage, access to quality health care), and demographic (e.g., educational attainment) influences confounds as permitted by their research design^2,21–24^.

In addition to detecting potential spillover effects, scholars also ought to consider *why* some people might be more likely than others to let their views toward the COVID-19 vaccine influence their views toward childhood vaccine mandates. In the context of longitudinal studies (strategy 1), this could take the form of asking people to self-report their attitudes toward vaccine safety and efficacy, and/or views toward government authority to impose vaccine mandates, at multiple points in time. Researchers could then examine the potential causes of COVID-19 vaccine spillover by (a) fashioning indicators of who adjusts their attitudes toward childhood vaccine mandates to match negative views toward COVID-19 vaccination (i.e., measures of who exhibits spillover effects), and (b) modeling the probability of expressing spillover effects as a function of change in vaccine confidence and/or attitudes toward government regulation.

Similarly, although observational methods (strategy 3) may not have the ability to observe change in opinion over time, researchers could nevertheless ask respondents to self-report how their views toward vaccine safety and/or government authority to mandate vaccination have changed over the course of the pandemic (recognizing the possibility of biased recall on this score). Researchers could then create interactive statistical models that assess the conditional effect of negative views toward COVID-19 vaccination on childhood vaccine policy attitudes, across self-reported change in vaccine confidence and mandate attitudes.

Likewise, scholars employing experimental methods (strategy 2) might consider directly manipulating the potential causes of vaccine spillover effects. For example, researchers could ask subjects to read short newspaper articles that present competing perspectives on arguments for/against vaccine mandates, prior to engaging with the types of experimental tasks described above. If those primed to think about arguments against vaccine mandates – particularly self-identified Republicans (who may be most amenable to arguments in favor of limited government) – are more likely to exhibit spillover effects than those presented with positive arguments, we may have reason to believe that changing views toward government authority to regulate vaccine behavior is responsible for spillover effects.

Finally, it is important to recognize that politicized COVID-19 vaccine spillover may be occurring not only in response to childhood vaccine mandates, but in other contexts as well. Attitudes toward vaccine mandates for adults in certain employment settings (e.g., requirements that healthcare workers vaccinate against influenza each year), as well as feelings toward elective vaccination (e.g., the decision to receive one’s annual flu shot) might also be influenced by politicized COVID-19 vaccine spillover. Researchers therefore ought to consider applying the methods described above toward studying this phenomenon in application to other vaccines and vaccine requirements.

In sum, researchers have three useful tools at their disposal for making an effort to document whether or not politicized views toward COVID-19 vaccines are shaping public support for childhood vaccine mandates more generally. Determining whether or not this is the case is vitally important, as it can help policymakers to preempt potential political challenges to policies encouraging universal childhood vaccination. I look forward to efforts to answer this question via a pluralistic set of methodological approaches.

## Supplementary information

Supplemental Information

## Data Availability

All data and syntax necessary to replicate Fig. 1 can be found at the following Open Science Framework page: https://osf.io/r95nk/.
