# Supplementary material for: Is partisan conflict over COVID-19 vaccination eroding support for childhood vaccine mandates?
Source: NPJ Vaccines. 2023 Feb 2;8:5. doi: 10.1038/s41541-023-00611-3 (PMC9892671; doi:10.1038/s41541-023-00611-3)
Supplement: Supplementary file 1 — Supplemental Information [file 41541_2023_611_MOESM1_ESM.pdf]

Supplementary Materials for: Is Partisan Conflict Over COVID-19 Vaccination Eroding Support for Childhood Vaccine Mandates?

Last Updated: 10/20/2022

Contents

Section 1: Additional Information about Blue vs. Red State Operationalization **2**

Section 2: Outcome Variable Generation – Weekly *Per Capita* VAERS Reports for MMR and MMRV Averaged Across Blue and Red States from 1/2006 - 10/2022. **3**

Section 3: Overview of the Regression Discontinuity (RD) Procedure **4**

Section 4: Summary of Regression Discontinuity Results **6**

Table S1. Regression Discontinuity Analyses Assessing the Effect of Change in COVID-19 Vaccine Availability on Differences in Red vs. Blue State Reporting 6

Section 1: Additional Information about Blue vs. Red State Operationalization

I distinguish between Republican-leaning (red) and Democratic-leaning (blue) states by turning to the Cook Political Report's Partisan Voting Index (PVI) measure of state partisan lean. Cook calculates PVI by first determining how each state voted in the two most recent presidential election cycles. They then average those quantities together, giving additional weight to the most recent presidential election contest; such that 2020 election results are given three-times more consideration in the calculation of the final average. Finally, PVI takes the difference between each state-level score and the national average.

The resulting index tells us the degree to which the partisan lean of any particular state deviates from the national average. For example, a state earning a score of D+3 on Cook's PVI favors Democrats by three percentage points more than the nation as a whole.

To facilitate comparison across states, I calculate VAERS reporting trends (described in detail below) across a balanced set of Blue and Red states. The number of states selected for analysis is, on some level, arbitrary. However, I wanted the number to be large enough as to account for potential heterogeneity across states, without being so large that it would include states that do not have a discernable partisan lean vis-a-vis the national average. Correspondingly, I include 16 states (approximately one third; 32%) in these analyses; i.e., the 8 states with the strongest PVI Republican lean, and the 8 states with the strongest PVI Democratic lean.

Section 2: Outcome Variable Generation – Weekly *Per Capita* VAERS Reports for MMR and MMRV Averaged Across Blue and Red States from 1/2006 - 10/2022.

The primary outcome variable in these analyses is (in broad terms) a measure of adverse events reported to VAERS logged across Blue vs. Red states. Specifically, I measure the *per capita* weekly reports pooled across Blue and Red states for the two most commonly administered forms of vaccines against Measles, Mumps, and Rubella; MMR and MMRV (which also inoculates against varicella; see: https://www.cdc.gov/vaccines/vpd/mmr/hcp/vacopt-factsheet-hcp.html).

I construct this measure by first summing up the total number of weekly VAERS reports (from January, 2006 - October, 2022) logged for the MMR plus MMRV vaccines across *each* of the eight blue and red states (sixteen states in total). Note that I selected the January 2006 starting point as it represents the first year in the series (VAERS data span from 1990-2022) in which both the MMR and MMRV vaccines were approved for public use.

I then divide each of these sixteen quantities by the total number of people living in each state, to account for the possibility that differences in population size might influence any differences in reporting I might observe across Blue vs. Red states. This creates sixteen *per capita* measures of VAERS reports for the two vaccines.

Next, I calculate average the total number of weekly reports to VAERS issued across the eight blue and eight red states (respectively). These two quantities serve as the outcome variables presented in Figure 1 in the main text.

Note, however, that in order to estimate the regression discontinuity analyses described in the previous section, I must work with a *single* outcome variable that denotes the difference between these two quantities. Correspondingly, I subsequently subtract the red state weekly averages from the blue state weekly averages to arrive at the final outcome variable featured in the regression discontinuity analyses.

Section 3: Overview of the Regression Discontinuity (RD) Procedure

To assess whether or not the trends documented in Figure 1 are not just substantively but *statistically* differentiable from one another, I employ a technique known as regression discontinuity (RD) analysis. RD allows me to determine whether or not the difference in weekly VAERS reporting averages in blue states vs. red states was significantly higher *following* the availability of a vaccine for COVID-19, relative to before it. If people living in Red States were comparatively more likely than those in Blue States to log reports to VAERS following the availability of COVID-19 vaccines, we may have reason to suspect that politicized vaccine spillover is indeed occurring.

The RD approach requires me to make several important assumptions and methodological decisions. Perhaps most importantly, RD requires selecting a date that differentiates between the (pre/post) periods denoting COVID-19 vaccine availability.

I select the first full week of March 2021 as this cut-off point, as March represents the first month in which some (but not all) US states began making COVID-19 vaccines available beyond select at-risk populations, and to the public more generally. This time frame serves as a "sharp" cut-off point in the RD analyses. While imperfect, this cut-off point has the benefit of offering a relatively-conservative assessment of potential spillover effects attributable to the availability of a COVID-19 vaccine; as it estimates those effects in a period where not all US adults had access to the vaccine.

Of course, and as alluded to above, I recognize states varied in the amount of time it took to make vaccines available to the general public. Correspondingly, I re-estimate all RD models to include a "fuzzy" cut-off point that treats the beginning of March *through* the beginning of April as differentiating between the two periods. This allows me to account for asymmetries in state level vaccine roll out when calculating the effect of vaccine availability on MMR VAERS reports.

Having defined suitable cut-off dates for the RD analyses, the procedure also requires making assumptions about the functional form of the data. Lacking any clear indication of quadratic or cubic functional forms from the scatter plots presented in Figure 1, I opted to fit linear trend lines to the pre/post-vaccine availability data. For robustness, I provide both sharp and fuzzy RD results that include a quadratic bias correction term.

Because I aggregate reports at the weekly level, 789 weeks are included in the estimation of the pre-COVID vaccine availability trends, while 82 are included in the post-vaccine period in the sharp RD tests. In the fuzzy RD tests – which, as mentioned above, span 8 weeks and include a cutoff point corresponding to the midpoint of that span – there are 793 observations in the pre-vaccine period, and 78 in the post-vaccine period. I estimate these models using the `rdrobust` command in `Stata 15`.

Note also that, for ease of interpretation – i.e., because relatively few members of the US population

log adverse event reports at any given time – I multiply the difference in *per capita* weekly reports across Red and Blue states (described in the preceding section) by a factor of 10^6.

Section 4: Summary of Regression Discontinuity Results

Table S1 presents the results of the RD procedures described above. Please see the caption accompanying this table for additional information about each of its entries.

The results present robust evidence in favor of the idea that red states logged comparatively more weekly MMR+MMRV VAERS reports *per capita* than blue states, in the post COVID-19 vaccine availability period (relative to before it). Specifically, both of the sharp RD procedures produce positively signed coefficients (indicating higher levels of VAERS reporting in red vs. blue states) that approached conventional levels of statistical significance at the $p < 0.10$ level, *two-tailed.* I also find that both conventional and bias-corrected fuzzy RD estimates produce positively signed coefficients, although this difference only attains conventional levels of two-tailed significance at the $p < 0.05$ level in the former case. Taken together, I detect evidence consistent with politicized COVID-19 vaccine spillover in three out of four statistical tests.

Table S1. Regression Discontinuity Analyses Assessing the Effect of Change in COVID-19 Vaccine Availability on Differences in Red vs. Blue State Reporting

Polynomial	Cutoff Type	Bias Correction	β	p	Evidence of Spillover?
Order 1 (Linear)	Sharp	Conventional	0.14	0.08	YES*
Order 1 (Linear)	Sharp	Quadratic	0.13	0.09	YES*
Order 1 (Linear)	Fuzzy	Conventional	0.74	0.04	YES
Order 1 (Linear)	Fuzzy	Quadratic	0.16	0.66	NO

Note. Results from four RD analyses presented; please see the preceding section for additional information about each estimation strategy (i.e., columns 1-3), and the assumptions they make. Column 4 (β) corresponds to the difference in Red versus Blue state reporting in the pre/post vaccine availability periods, with a corresponding p value presented in Column 5. Column 6 offers an assessment of whether or not the available evidence provides evidence of politicized vaccine spillover. This is the case in 3 out of 4 tests, with the caveat that asterisked entries in this column refer to instances in which the associated p value approached conventional levels of statistical significance at the $p < 0.10$ level, two-tailed.
